# Updates on Endoscopic Stenting for Unresectable Malignant Hilar Biliary Obstruction

**DOI:** 10.3390/jcm13185410

**Published:** 2024-09-12

**Authors:** Tadahisa Inoue, Itaru Naitoh

**Affiliations:** 1 Department of Gastroenterology, Aichi Medical University, 1-1 Yazakokarimata, Nagakute 480-1195, Aichi, Japan; 2 Department of Gastroenterology, Nagoya City University Midori Municipal Hospital, 1-77 Shiomigaoka, Midori-ku, Nagoya 458-0037, Aichi, Japan; inaito@med.nagoya-cu.ac.jp

**Keywords:** malignant hilar biliary obstruction, plastic stent, metal stent

## Abstract

Malignant hilar biliary obstruction (MHBO) can cause obstructive jaundice and/or cholangitis necessitating appropriate biliary drainage. Endoscopic biliary stenting is the first-choice treatment, especially in unresectable cases, owing to its minimally invasive nature and utility. However, the hilar region is complex because of the branching and curving of bile ducts, making strictures in this area more complicated. Therefore, MHBO stenting is challenging, and treatment strategies have yet to be established. Furthermore, recent advances in antitumor therapies have altered the background surrounding the development of stenting strategies. Therefore, it is necessary to understand and grasp the current evidence well and to accumulate additional evidence reflecting the current situation. This study reviews the current status, issues, and prospects of endoscopic stenting for MHBO, especially in unresectable cases.

## 1. Introduction

Malignant hilar biliary obstruction (MHBO) is caused by various primary diseases and conditions including cholangiocarcinoma, gallbladder cancer, pancreatic cancer, liver metastasis, peritoneal dissemination, and lymph node metastasis. MHBO can cause obstructive jaundice and/or cholangitis necessitating appropriate biliary drainage. Biliary drainage is effective not only in improving symptoms, but also in enhancing the prognosis and quality of life of patients. Among the methods of biliary drainage, endoscopic biliary stenting is the first-choice treatment, especially for unresectable cases, because of its minimally invasive nature and utility. However, the hilar region is particularly complex because of the branching and curving of bile ducts, making strictures in this area more complicated. Consequently, the situation varies widely depending on the location, range, and branching pattern of the strictures, and the technical difficulty of drainage in MHBO is significantly greater than that in the treatment of malignant distal biliary obstruction. Therefore, the management of MHBO is challenging, and treatment strategies are yet to be established.

In this review, we summarize the current status of and challenges facing endoscopic stenting for MHBO, with particular focus on unresectable cases.

## 2. Drainage Area

In MHBO, the left and right intrahepatic ducts can be separated depending on the stricture location, which is usually categorized using the Bismuth classification; the Bismuth classification is a classification system based on the extent of ductal infiltration. Thus, the better option between inserting a single stent into the left or right lobe (unilateral stenting) and using multiple stents for bilateral stenting remains unclear. Although draining all regions is physiologically preferable, bilateral multiple stenting is technically more challenging both during the initial placement and reintervention in the event of stent occlusion. Additionally, sufficient drainage can be achieved with unilateral stenting alone in some cases, making this topic controversial.

Three retrospective studies [1,2,3] have evaluated the association between drained liver volume and drainage effectiveness in patients with unresectable MHBO. Vienne et al. [1] have reported that the most important factor associated with drainage effectiveness was a drained liver volume of >50%. Additionally, >50% drainage was associated with a longer median survival (119 days and 59 days, *p* = 0.005). Takahashi et al. [2] used computed tomography volumetry to measure the drainage area, and multivariate analysis indicated that the drained liver volume is an independent factor contributing to the effectiveness of drainage (odds ratio 2.92, 95% confidence interval 1.648–5.197, *p* < 0.001). Receiver operating characteristic analysis for effective drainage showed cutoff values of 33% for liver volume in patients with preserved liver function (normal liver or compensated liver cirrhosis) and 50% for patients with impaired liver function (decompensated liver cirrhosis). Additionally, drainage of the atrophic liver lobe was associated with drainage-associated cholangitis. Morimoto et al. [3] evaluated patients who received systemic chemotherapy using a three-dimensional image volume analyzer to evaluate the liver drainage area. Patients with >80% drainage had significantly longer survival than those with <80% drainage (median 450 days and 224 days, *p* = 0.0033). Moreover, multivariate Cox proportional hazards regression analysis revealed >80% liver drainage as a significant prognostic factor for overall survival (hazard ratio 0.35, 95% confidence interval 0.20–0.62, *p* = 0.0003).

Two randomized controlled trials (RCTs) [4,5] compared unilateral and bilateral drainage in patients with unresectable MHBO. Regarding the use of plastic stents (PSs), De Palma et al. [4] have reported that unilateral placement has a significantly higher rate of successful stent insertion than bilateral placement (88.6% and 76.9%, *p* = 0.041), whereas bilateral placement has a significantly higher rate of complications than unilateral placement (26.9% vs. 18.9%, *p* = 0.026). However, this RCT was published in 2001 and may not be appropriate for application to the present day because treatment devices have made great advances since then. Lee et al. [5] conducted an RCT using uncovered metal stents (MSs) in 2017. No significant difference in technical success rates (95.5% vs. 100%, *p* = 0.244) were observed between the two placement types, whereas the clinical success rate was significantly higher in bilateral placement than in unilateral placement (95.3% vs. 84.9%, *p* = 0.047). Additionally, the cumulative stent patency duration was significantly longer in bilateral placement than unilateral placement (median 252 days and 139 days, *p* < 0.01). Bilateral placement was also identified as a favorable factor for stent patency by the multivariate Cox proportional hazard model (hazard ratio 0.30, 95% confidence interval 0.172–0.521, *p* < 0.001). Furthermore, bilateral placement was positively associated with survival in the multivariate analysis (hazard ratio 0.415, 95% confidence interval, 0.259–0.666; *p* < 0.01).

Based on these results, the treatment approach for unresectable MHBO is fundamentally based on bilateral stenting. However, owing to the highly variable nature of MHBO in terms of location and extent, a drainage volume-based approach may be more suitable in clinical practice than simply deciding between unilateral and bilateral stenting. Although only retrospective studies have assessed drainage volume, securing a larger drainage area may be desirable, particularly in cases involving chemotherapy [3,6]. However, placing a stent in an atrophic lobe may induce unnecessary cholangitis [2] and is not always recommended. However, the threshold for defining atrophy remains unclear; further prospective studies are needed to clarify the appropriate drainage volume and strategies.

## 3. Stent Type Selection

### 3.1. Uncovered Metal Stent

Stents are classified into PSs, uncovered MSs, or covered MSs; however, PSs and uncovered MSs have traditionally been used in MHBO because covered MSs can obstruct the side branch of the intrahepatic bile ducts.

Two RCTs [7,8] have compared uncovered MSs and PSs in a large population of patients with unresectable MHBO (Table 1). Mukai et al. [7] have reported that uncovered MSs have a significantly longer stent patency (median 359 days and 112 days, *p* = 0.0002), and the number of re-interventions is significantly lower for uncovered MSs (mean 0.63 times and 1.80 times, *p* = 0.0008). Additionally, the overall total cost of drainage treatment was significantly lower in patients who underwent uncovered MS placement (*p* = 0.0222). Sangchan et al. [8] have reported that the successful drainage rate is significantly higher with uncovered MSs (70.4% and 46.3%, *p* = 0.002). Additionally, the median stent patency (103 and 35 days, *p* < 0.001) and median overall survival (126 days and 49 days, *p* = 0.002) were both significantly longer with uncovered MSs. However, the participants in these two studies mainly included patients who underwent unilateral placement.

Although this was a retrospective study, Xia et al. [9] recently compared bilateral PS placement and bilateral uncovered MS placement in a large population of patients with unresectable MHBO. They have reported that bilateral MS placement has a significantly higher clinical success rate (99.0% and 71.9%, *p* < 0.001), longer median symptom-free stent patency (9.2 months and 4.8 months, *p* < 0.001), and fewer total interventions (1.3 and 2.0, *p* < 0.001). Additionally, the median survival was also significantly longer in bilateral MS placement than in PS placement (7.2 months and 4.1 months, *p* = 0.015).

Based on these results, uncovered MSs may have higher clinical success rates, longer stent patency, fewer number of interventions, and lower total costs, as well as longer survival durations. Therefore, uncovered MSs have long been recommended for unresectable cases, especially for patients with a prognosis of ≥3 months [10,11]. However, the application and recommendations for uncovered MSs remain debatable.

### 3.2. Plastic Stent

Uncovered MSs are not removable and pose difficulties in reintervention. The success rate of reintervention for stent occlusion in patients who underwent bilateral MS placement is insufficient at 76.3–85.7% [12,13,14,15,16]. The expected survival duration of patients with MHBO, including those with cholangiocarcinoma, is currently increasing with advancements in antitumor therapy [17,18]. Therefore, approximately half of patients can experience stent occlusion even if bilateral uncovered MSs are placed. Additionally, some cases in which antitumor therapy is significantly effective and conversion surgery can be adapted have also been reported. Therefore, in many institutions, PSs are now commonly selected over uncovered MSs in patients with an expected longer prognosis or those who are likely to respond to antitumor therapy. However, PSs have a short stent patency period; consequently, some researchers have attempted to place them above the papilla (the distal stent edge is located within the bile duct) [19,20] in order to obtain a longer stent patency duration while keeping the benefits of PSs including easy removability.

Two RCTs [19,20] investigated PS placement above the papilla in patients with unresectable MHBO (Table 1). Kurita et al. [19] reported that the median cumulative stent patency was longer in the above the papilla placement than the conventional placement across the papilla (123 days vs. 51 days, *p* = 0.031), whereas the technical success rate, clinical success rate, reintervention rate, adverse events, and survival probability did not differ between the two groups. Kanno et al. [20] compared PS placement above the papilla and uncovered MS placement. They reported that the technical success rate (100% vs. 96.6%, *p* = 1.00), clinical success rate (90.0% vs. 88.9%, *p* = 1.00), adverse event rate (10.5% vs. 15.2%, *p* = 0.75), and time to recurrent biliary obstruction (median 250 days and 361 days, *p* = 0.34) were not significantly different between the PS placement above the papilla and MS placement groups.

Based on these studies, PS placement above the papilla may be a better option than their placement across the papilla and may be a viable alternative to uncovered MSs, especially when considering reintervention. However, the evidence is limited, and further studies are needed to evaluate the true benefits of this method.

### 3.3. Covered Metal Stent

Covered MSs were originally developed to prevent tumor ingrowth and have the advantage of removability such as PSs. Covered MSs are commonly used for malignant distal biliary obstruction, whereas covered MSs are not commonly used for MHBO because they occlude the side branches of the intrahepatic bile ducts. Recently, a slim-covered MS with a diameter of 6 mm which is thinner than the conventional 8 mm or 10 mm diameter MSs, has emerged [21]. This novel stent is intended to reduce the risk of side-branch obstruction, to be removable like PSs, to make reintervention straightforward, and to achieve longer patency than PSs in MHBO stenting.

Only one RCT by Paik et al. [22] evaluated slim-covered MS, comparing bilateral slim-covered MS and PS placements in patients with unresectable MHBO (Table 1). The mean time to recurrent biliary obstruction was significantly longer with the slim-covered MSs than with the PSs (190 days and 96 days, *p* = 0.02), and the rates of adverse events, including segmental cholangitis, were similar between the two groups, although stent migrations were more frequent with the slim-covered MSs. However, in this study, all stents were placed across the papilla. According to four retrospective studies [21,23,24,25] regarding slim-covered MSs, placement above the papilla demonstrated a longer patency period, similar to that of PSs. Another issue to consider with this treatment is the existence of partially covered and fully covered MSs. Because the removability rate for reintervention was 100% in fully covered MSs and 46–67% in partially covered MSs [21,23,24,25], using a fully covered MS would be better to obtain removability performance.

Based on the currently available evidence, slim-covered MSs may be a useful option for MHBO, although the evidence is limited. Further prospective studies are needed, including an investigation of their placement above the papilla and comparisons with uncovered MSs.

**Table 1:** Randomized controlled trials for stent type selection for unresectable malignant hilar biliary obstruction.

Author	Drainage Treatment	No. of Patients	Bilateral Placement	Technical Success		Clinical Success		Procedure Related-Adverse Event		Median Stent Patency		Median Overall Survival	
Mukai et al., 2013 [7]	PS	30	50%	-	-	-	-	-	-	112 d	***p* = 0.0002**	188.5 d	*p* = 0.2834
	Uncovered MS	30	53.3%	-		-		-		359 d		219.5 d	
Sangchan et al., 2012 [8]	PS	54	0	85.2%	*p* = 0.792	46.3%	***p* = 0.011**	40.7%	*p* = 0.102	35 d	***p* = 0.000**	49 d	***p* = 0.0021**
	Uncovered MS	54	0	83.3%		70.4%		25.9%		103 d		126 d	
Kurita et al., 2022 [19]	PS (across the papilla)	22	27.3%	100%	*p* = 0.488	100%	-	18.2%	*p* = 0.108	51 d	***p* = 0.031**	194 d	*p* = 0.452
	PS (above the papilla)	21	28.6%	95.2%		100%		14.3%		123 d		293 d	
Kanno et al., 2023 [20]	Uncovered MS	46	82.6%	95.7%	*p* = 1.00	88.9%	*p* = 1.00	15.2%	*p* = 0.75	361 d	*p* = 0.34	232 d	*p* = 0.11
	PS above the papilla	38	86.8%	100%		90.0%		10.5%		250 d		215 d	
Paik et al., 2023 [22]	PS	25	100%	100%	*p* > 0.99	76%	*p* = 0.27	32%	*p* > 0.99	96 d *	***p* = 0.02**	-	-
	Slim-covered MS	25	100%	100%		88%		32%		190 d *		-	

PS, plastic stent; MS, metal stent. Bold values indicate statistical significance (*p* < 0.05). * Mean values.

## 4. Future Challenges

### 4.1. Three or More Stent Placements

As previously mentioned, a previous study has suggested that securing a drainage liver volume of >80% is preferable, especially in patients receiving chemotherapy [3]. Physiologically, increasing the drainage volume is always beneficial. However, achieving >80% drainage frequently requires the placement of three or more stents. In recent years, advancements in devices, including stents, have made the placement of three or more stents more feasible than before [26,27]. However, placing three or more stents further increases the technical difficulty, necessitating the clarification of whether such a procedure is truly necessary and, if so, determining the optimal method and technique for placement.

### 4.2. Combination of Local Biliary Therapy

Several local biliary treatment methods such as radiofrequency ablation [28,29] and photodynamic therapy [30,31,32], have been introduced in combination with stent placement for unresectable MHBO. These treatments potentially extend stent patency periods and improve survival, particularly in cases of extrahepatic cholangiocarcinoma. Although these methods theoretically have significant potential utility, their efficacy remains unclear as previous studies have reported conflicting results [33]. Additionally, the devices currently available for local biliary therapy are not necessarily well suited for treating biliary strictures [34]. Therefore, these treatments are currently being clinically investigated. Further advancements of these devices and additional studies are required.

### 4.3. Endoscopic Ultrasound-Guided Approach

As mentioned earlier, bilateral stenting is the standard approach for unresectable MHBO; however, reintervention after the placement of bilateral uncovered MSs is difficult and troublesome. To simplify this reintervention process and achieve longer stent patency, approaches involving endoscopic ultrasound-guided biliary drainage including hepaticogastrostomy to the left intrahepatic duct, hepaticoduodenostomy to the right intrahepatic bile duct, and bridging from the left to the right intrahepatic ducts, or a combination of endoscopic ultrasound-guided biliary drainage and conventional transpapillary drainage, have recently been attempted [35]. Currently, endoscopic ultrasound-guided biliary drainage is mainly used when the transpapillary approach is difficult, but it is also increasingly considered a first-line approach. Particularly with the combination method, the separate approach routes to the right and left lobes of the liver might overcome the limitations of the current conventional transpapillary bilateral stenting. However, evidence regarding this technique is also limited; therefore, further studies are required.

## 5. Conclusions

This review outlined the current status and challenges associated with the endoscopic stenting of unresectable MHBO. Stenting for MHBO is highly complex because of the anatomical intricacies and numerous factors involved; thus, an optimal method has yet to be established. Furthermore, recent advances in antitumor therapies have altered the background surrounding the development of stenting strategies, necessitating the accumulation of additional evidence reflecting the current situation. Moreover, new approaches, devices, and techniques are emerging, and a breakthrough method that truly benefits patients is also expected in the near future.

## Data Availability

Not applicable.
